# Stakeholder Perspectives on Barriers for Healthy Living for Low-Income African American Families

**DOI:** 10.3389/fped.2014.00137

**Published:** 2014-12-04

**Authors:** Veronnie Faye Jones, Michael L. Rowland, Linda Young, Katherine Atwood, Kirsten Thompson, Emma Sterrett, Sarah Morsbach Honaker, Joel E. Williams, Knowlton Johnson, Deborah Winders Davis

**Affiliations:** ^1^University of Louisville School of Medicine, Louisville, KY, USA; ^2^Pacific Institute for Research and Evaluation, Louisville, KY, USA; ^3^Clinical Psychology in the Family Therapy Program, Kent School of Social Work, University of Louisville, Louisville, KY, USA; ^4^Department of Pediatrics, Indiana University School of Medicine, Indianapolis, IN, USA; ^5^Department of Public Health Sciences, College of Health, Education and Human Development, Clemson University, Clemson, SC, USA; ^6^Pacific Institute for Research and Evaluation-Asheville, Asheville, NC, USA

**Keywords:** childhood obesity, qualitative methods, poverty, African American, United States

## Abstract

**Background:** Childhood obesity is a growing problem for children in the United States, especially for children from low-income, African American families.

**Objective:** The purpose of this qualitative study was to understand facilitators and barriers to engaging in healthy lifestyles faced by low-income African American children and their families.

**Methods:** This qualitative study used semi-structured focus group interviews with eight African American children clinically identified as overweight or obese (BMI ≥ 85) and their parents. An expert panel provided insights in developing culturally appropriate intervention strategies.

**Results:** Child and parent focus group analysis revealed 11 barriers and no definitive facilitators for healthy eating and lifestyles. Parents reported confusion regarding what constitutes nutritional eating, varying needs of family members in terms of issues with weight, and difficulty in engaging the family in appropriate and safe physical activities; to name a few themes. Community experts independently suggested that nutritional information is confusing and, often, contradictory. Additionally, they recommended simple messaging and practical interventions such as helping with shopping lists, meal planning, and identifying simple and inexpensive physical activities.

**Conclusion:** Childhood obesity in the context of low-resource families is a complex problem with no simple solutions. Culturally sensitive and family informed interventions are needed to support low-income African American families in dealing with childhood obesity.

## Introduction

Childhood obesity rates have increased to alarming rates in the United States. Almost 32% of children ages 2–19 years are overweight or obese (1). Rates vary by geographic location and by race/ethnicity and gender (2). Childhood obesity is a risk factor for a variety of negative physical and psychosocial consequences (3, 4). Studies have shown an association between obesity and cardiovascular disease, hypertension, metabolic syndrome, sleep apnea, non-alcoholic fatty liver disease, asthma, and orthopedic problems (5). Some common psychosocial problems may include a higher prevalence of depression and lower health-related quality of life, social discrimination, and low self-esteem (5). In addition to health risks associated with obesity, there is also a tremendous economic impact on individuals and society. The annual cost of obesity-related expenditures is approximately 10% of all medical costs and it is projected that this number could reach 16–18% by 2030 (1). Medical expenditures together with lost productivity have been estimated to be $254 billion for adolescents alone (1). These costs will continue to escalate as chronic diseases progress into more debilitating stages.

Prevention or early recognition and intervention in early childhood are key to reducing the long-term effects of being overweight or obese (6, 7). However, much remains unknown about the best methods for prevention and the effectiveness of specific interventions for various populations of children. A number of intervention studies have attempted to reduce the behavioral risk factors for childhood obesity (8). Many of these interventions have produced only small changes in targeted behaviors and no significant changes in BMI compared to controls (9). A possible explanation for the lack of effect of these interventions is that the interventions do not incorporate the cultural context of the targeted population (10). In addition, the dynamic interaction between family processes and obesity needs to be further explored (11–14) and incorporated into the intervention strategies.

Previous investigators have emphasized the importance of the role of culture in developing interventions (10, 14–19). Unfortunately, cultural considerations may be overlooked when targeting minority youth. For example, many African American, Hispanic, and low-income youth and adults are more likely to underestimate their weight than their Caucasian and middle-income counterparts. Additionally, the role of over-eating as a culturally bound coping mechanism is also important to consider. For many ethnic groups, feeding family members is an expression of love. Food is central to family and social gatherings and refusal to eat a large amount of food is considered rude. Therefore, there is a need to put programs in place that are cognizant of these countervailing norms and attitudes and frame messages that focus on exercise and diet from the culturally sensitive perspective of health rather than weight.

Cultural sensitivity when creating health promotion programs or interventions, as it relates to health promotion research, should factor in ethnic and/or cultural characteristics, experiences, norms, values, behavioral patterns, and beliefs of specific groups as well as relevant historical, environmental, and social factors (15).

Beyond the role of culture, familial and social context is a significant influence on eating and exercise norms. As parents most often provide food and monitor food intake throughout childhood and early adolescence, they are important partners in reducing childhood obesity. Consistent with this, interventions that target both parents and children have been found to be more effective than those targeting children alone (8).

The purpose of this study is to understand factors that impact obesity for inner-city African American children and their families. An additional aim was to understand how the ideas of community health experts can be used to create a successful, culturally adapted healthy-lifestyle intervention for low-income minority families.

## Materials and Methods

### Participants

#### Parents and children

A convenience sample of parents and children (Table 1) was recruited from an urban academic pediatric primary care practice in the southern United States that serves approximately 6,100 patients birth through 18 years of age who are predominantly African American and Medicaid recipients. In a random surveillance of medical records, approximately 50% of the children between 6 and 10 years of age were found to have a BMI equal to or in excess of the 85th percentile. Therefore, we targeted African American families with children between the ages of 6–10 years of age who were patients at the clinic with a BMI of >the 85th percentile. Parents or guardians visiting the clinic over a 4-month period were given a brochure that explained the study and then asked to complete a form if they were interested in being contacted to participate in a healthy lifestyles focus group.

**Table 1:** **Description of participants**.

Participant type	Sample size	Characteristics
Caregivers	9	High school education
		Income
		4 < $15,000
		3 $15,000–$25,000
		2 > $35,000
		Medicaid recipients
		Age 25–70 years
		Gender
		8 Females
		1 Male
Children	8	Age
		2–8 years
		4–9 years
		2–10 years
		Gender
		6 Females
		2 Males
Expert panel members	9	Professional association
		3 Medical Doctors
		1 Psychology
		1 Registered Nurse
		4 Community Leaders
		Race/ethnicity
		6 African American
		3 Caucasians

#### Expert panel

A nine-member expert panel (EP) (Table 1) was recruited using a purposive sampling strategy. The EP included three physicians, one psychologist, one registered nurse, and four local community members who represented the local YMCA and health department. All have significant experience working with low-income African American families.

### Procedures

#### Parent and child focus groups

Participants were then scheduled for a focus group that was convenient for them. Parent focus groups were based on the child’s age due to varying stages of development (6–7 and 8–10 years). Parents were asked if they would like to receive additional calls as reminders when they were scheduled for a focus group, and most wanted multiple phone reminders. If participants did not show up to their scheduled focus group, they were called for participation in a subsequent focus group. The majority of participants that did not show up at a scheduled time, had transportation or a childcare emergency that kept them from attending. However, some did not respond to additional contact attempts after asking to be rescheduled for another focus group. Participants were called at various days and times of the day according to their listed availability. Telephone numbers were obtained for both parents and additional family members in case there was an issue with a number changing or a phone being turned off. In total, 27 caregivers signed up for the focus groups. Twenty-five caregivers (93%) were able to be reached via phone. Twenty caregivers (74%) scheduled themselves and one of their children for a focus group. As an incentive, each participant (both parent and child) was given a $25.00 gift card for attending the focus group session. Childcare services and healthy snacks were also provided during the focus groups and the location of the meeting was held across the street from a common bus route.

The focus groups included dyads of a parent and a child, which were conducted separately. Only one child per family was recruited. A total of three focus groups were conducted with children from 8 to 10 years of age, and three focus groups with their parents or guardians. The focus group interviews were conducted on Saturday afternoons during the months of January through March 2012. This study was approved by the University Institutional Review Board.

Separate focus group moderator guides were developed by the authors for parents and children. The focus groups were conducted to explore thoughts, feelings, experiences, associations, language, assumptions, and barriers that may facilitate or impede implementation of healthy lifestyles, primary care-based intervention. We use the term “parents” to refer to both the parents and guardians in the group.

There was one experienced facilitator for each of the three parent and child groups along with two note-takers for each children’s group and one note-taker for each parent group. A child psychologist was present during and after each of the children’s focus groups. Focus group sessions lasted 90–120 min and were audio-recorded and transcribed for analysis. At the conclusion of the focus groups, the height and weight of both parent and child were recorded to determine BMI.

Focus group questions were designed to gather in-depth information regarding what is considered a healthy lifestyle, what are the barriers to involvement in obesity prevention interventions, messaging relating to diet and physical activity, and the recruitment/retention of families to participate in an obesity-focused program. Additional questions used as probes were asked to determine the perceived effectiveness of various delivery methods such as a face-to-face program, use of social media, mobile technology, and/or written materials.

#### Expert panel of key informant focus groups

The second phase of our study included two focus group discussions with an EP of community leaders who reflected on the themes generated from the parent and child focus groups and made suggestions and recommendations for a possible healthy-lifestyles intervention and other ways to help combat childhood obesity. Two separate focus groups were conducted to accommodate the members of the EP. The authors developed a separate focus group moderator guide for the EP. Prior to the EP focus group discussion, each member of the panel was given a copy of the themes and associated comments from the parent and child focus groups in order to familiarize themselves with the prior data collection results. These focus group sessions were also audio-recorded and transcribed for later analysis.

### Data analysis

In an effort to better understand the context and analysis of the data, we present both the parents’ and children’s results collectively followed by the EP results. The qualitative analysis revealed 13 key themes related to the parent and child focus group discussions and three key themes for the EP.

The transcripts from the audio-recorded focus groups were compared with the tapes and with the investigators notes taken during the discussions to check for completeness and accuracy of the data. The investigators met following each focus group and reviewed the procedures noting any strengths or weaknesses in the focus group procedures. Four of the investigators served as coders. Each coder analyzed the data and identified themes separately. The primary investigator used QDA Miner^®^ V3, a mixed methods software program to organize and code frequencies of the themes and patterns found among the coders. The software also allowed the primary investigator to create a list of variables that was used to conduct specific searches of the coded text. The themes were shared with each of the coders until consensus was reached regarding each of the themes. The results are presented according to the themes that emerged from the interview data.

## Results

### Parent and child focus group results

#### Healthy/unhealthy eating

One emerging theme was that parents indicated they were confused about healthy food choices because information about nutrition often changes. Even reading nutrition labels on food items was reported to not be helpful. The following remarks are typical of this general sense of confusion.

I never read [nutrition labels]. I don’t understand [them]. I try to look at the number of calories and how much sugar is in it; my kids try to read the labels too.

People are telling me different things; too many tomatoes are bad, too much fruit becomes sugar. I don’t know what to believe.

There was a consensus among both parents and children that fruits, vegetables, and turkey meat are primarily healthy food choices. Both groups were also aware that foods like pizzas and sugary drinks (such as sodas, juices) were unhealthy food options. However, all parents commented on the difficulty they encounter in getting their children to eat healthier food options. One reason cited by parents why they and their children disliked some of the healthier food options is because those foods lack the flavor that is so characteristic of traditional African American cooking. The parents were aware of the high sodium in their diet, but they indicated that they are accustomed to this taste and found it difficult to change and this change came with a financial cost.

Parents who were on some form of financial assistance (e.g., WIC, food stamps) felt they were limited in what foods they could buy. One mother said, “I get WIC and thought it was supposed to be healthy stuff, but it’s not. I got tired of trying to figure out what was healthy and what wasn’t.” Another said, “I can only get sweet potatoes on WIC. They give you a $7.00 voucher for fruits and vegetables.” And a third parent added, “With the amount of food stamps I get, I can’t buy the more expensive olive oil. It is better, but it costs more.”

Often there was conflicting information and statements from the children’s focus groups compared with what their parents said during their interviews. For instance, the children generally believed that it is more difficult to eat healthy foods at home, but that it is much easier to eat healthier foods outside of the home because there are healthier options available. Overall, the children believed fruits and vegetables were healthy foods. Yet, in one of the children’s focus groups, when asked about food choices eaten in the last 24 h (dinner and breakfast), most indicated they had eaten fried foods, and no one mentioned having eaten fresh fruits or vegetables.

#### Fast foods

We asked the parents and children about eating at fast food restaurants and found that most of the parents indicated they eat fast food. Some of the parents said they use fast food because it is a convenient option and sometimes used it as a reward for their children. One parent stated:

We live by a bunch of restaurants and that’s what they want to eat. When I get off work, that’s probably what they are going to get, some chicken nuggets and some fries … We eat twice a week at fast food restaurants.

Buffets were also mentioned as a popular option for dining out. One parent reasoned that, “because they have the buffet and [the children] can eat all they want.” Another parent commented that her 2-year old child has french fries every day because he is a “potato baby.” Yet another parent said she did not allow her children to eat a lot of fast foods. She stated:

My kids don’t like me very much because I won’t buy them (unhealthy) stuff just because somebody else does … I don’t do fast food. They do go there when they are with their grandma. She used to cook everything [herself] but now to make her life easier, she takes her grandkids to get fast food.

Parents professed they are “finicky” eaters themselves, and that their finicky eating habits had an effect on their children. Some parents would not offer or cook certain healthy foods in the home because they themselves did not like the healthier food options. One parent noted, “My eating habits are atrocious and since I’ve had custody of my granddaughter, her eating habits have become atrocious.”

#### Balancing family needs

Another emerging theme was the difficulty experienced when parents were trying to balance the nutritional needs of their obese child with the food desires of the other children or relatives in the home. Several parents indicated they also had a child with a diagnosed condition of ADHD with a high metabolism, while others in the household were experiencing weight problems. This sometimes created confusion and tension in the home around rule setting and food access. The obese child’s food choices were monitored and limited while the food choices of other children were not. One parent remarked:

My son is ADHD. So, with his metabolism, he can eat whatever. It ain’t going to affect him, but my daughter is the one that it affects. But she will see him eat it … but then I tell him, you can’t eat what you want to eat in front of her. It’s a fight going on. So that is why I started locking food up. But my son gets up in the middle of the night and sneaks. He craves sweets. Now, my daughter, she will try to do it when I am asleep … . She will try to go get her a bag of popcorn. I smell it and hop up. I’ll go, “What are you doing?” But kids, they don’t think about the after-effect.

Reflecting on these conflicting messages, one child explained that only she was required to ask permission to eat unhealthy food items, while everyone else in her family had greater access to them. She said: “I have to ask to eat them.” Many parents viewed the denial of food items to their children as denying their children something that they want, and they felt uncomfortable, sometime unwilling to do that.

#### Family rules and eating

In general, most participants indicated they did not have specific rules about eating in their homes. For instance, eating dinner together as a family seemed to be the exception, rather than the rule. Due to work schedules and after-school activities, most of the parents and children reported they do not eat together as a family. The children recalled many rules about eating at home, such as having to eat all their vegetables, not eating in the middle of the night, asking permission before having a snack, and having to finish everything on their plate before dessert. Sometimes there were inconsistent or contradictory rules in the home where rules about eating where established by the parent but ignored by other extended adult family members. One child commented “Sometimes my mom tells me to eat at the table, but my granny says I can go eat in her room and watch TV with her.”

#### Physical activity

We asked the parents and children to comment on the level of physical activity they thought was appropriate. Overall, the parents did not seem to understand how much physical activity is needed or recommended for children or adults. In one of the adult focus groups, the parents indicated that children need less exercise than adults do. All of the parents stated their children would like for them to participate in some activity with them, however, many of the parents stated they have other obligations and busy schedules, while some simply offered excuses for not participating in family physical activities, such as being too tired, not interested in physical exercise, or being too old.

Parents were extremely concerned about their children doing physical activities outdoors in their neighborhoods, as they contended their neighborhoods are unsafe and did not allow their children to play outside. Many of the parents also considered the parks in their area of town as unsafe. One mom stated,

Like when I was little, you could go out and ride your bike to lose weight. Nowadays, it’s not that free anymore for kids. You just can’t say, “Go ride your bike with your friends.” You can’t do that now.

While the majority of parents stated they do not let their children go and play at the park, many of the children stated they do go to the park and play. One child said, “Because there is a park near my house. So I just walk there and just go swing on the swings.”

#### Indoor activity

Another common theme was that their children’s free time involved activities on the computer that included playing video games, most of which do not promote physical movement. Most of the parents indicated they had a Wii™ game system at home, but consistently stated it was not helpful in keeping their children physically active. They stated that the children were able to play the games sitting down or that their interest in playing with the Wii™ decreased over time. Another parent voiced concern over her son’s excessive use of the computer.

Although many of the children in the study indicated they play video games for diversion, all of them reported that they get enough physical exercise but had difficulty giving concrete examples of their activities. Most of the children seemed interested in participating in school sports; however, only a few indicated actually being involved in structured sports activities or teams.

Participants in both parent and child focus groups indicated that participation in YMCA activities would be helpful. Some of the participants reported they have a membership to the YMCA, while others spoke of not being able to get a YMCA membership for their child because children have to be at least 9 years of age to participate in YMCA activities. One child who did have a YMCA membership stated, “Usually we go to the YMCA, but usually my mom doesn’t have time, so we only went to the YMCA two times [this year]. At the YMCA they have a lot of things [to do].”

#### Sleep and obesity

When the children were asked about sleep and health, most agreed that sleep was important for health but did not directly connect sleep with eating behaviors. Many consistently reported that their bedtimes were between 8:30 and 9:30 p.m. on weeknights; but all commented that they did not have a specific bedtime on weekends. Some claimed to never have a bedtime. One child commented, “I can go to my room but I don’t have to [go to] sleep. On the weekends … I make up my own bedtime. Whenever I fall asleep.” Yet many of the participants seemed to draw a connection between being rested and having enough energy to exercise. However, the parents overwhelmingly mentioned bedtime in the context of allowing them to have free time once their children went to bed.

#### Causes of childhood obesity

There were a range of beliefs among the parents regarding the cause of their child’s obesity. Many of the parents believed their child’s obesity struggles were either the fault of the child, or their own fault, or as one parent believed, society was partly at fault. She stated:

The world helped me with my daughter getting big. People thought she was cute when she was chubby, but y’all helped do this by giving her cookies, etc …

Some parents justified their child’s weight issue as being from a “big family” and that the child was never going to be small, but the parents did want the child to be healthy. Many of the parents did not understand how physicians determined who was obese or overweight. They expressed confusion about the relationship between height, weight, and clothing size and not understanding how to determine what their children should weigh. The parents faced constant criticism about their children’s weight from health care providers, which they perceived as offering them little meaningful guidance.

#### Parents’ own struggles with weight

Although all of the parents were concerned about their children’s weight, only a few admitted to being obese themselves and even fewer admitted to being concerned about their own weight even when they were obese; yet they all expressed their wishes for their children to be healthy. A parent with custody of her granddaughter, who stated she was overweight herself, described how it makes you feel when people focus on your weight:

People don’t realize how it makes you feel when they say something about your weight. My mother is very mean about mine and [her granddaughter’s] weight. … [My mother says,] “I don’t know why you want to look like that”.

She further stated that her mother would not even take her granddaughter across the street because she doesn’t like the way she looks. She said, “I would never let myself look like that.”

Some parents tried to warn their children about the effects of having diabetes and having to take insulin shots daily. As one parent reported telling her child, “You don’t want to have to give yourself a shot in the belly. When you’re fat, people don’t want to be around you.”

The parents expressed helplessness about how to address issues about their weight or their child’s weight. Again, many parents justified their child’s weight problem and their own as being from a “big family.” They also communicated feeling like they had no control over their external environment. The issues of obesity, as well as other health-related problems such as high blood pressure and diabetes, were common among multiple immediate- and extended-family members. Some of the parents noted their own issues with stress, self-esteem, boredom, and laziness as causes for their weight problems.

Indulging in food is your stress reliever. Some people turn to alcohol; some to drugs, but most turn to food. Stressors are financial; feeling overwhelmed; losing my teeth; grief. My sister was murdered and I didn’t know how to grieve. There was food all the time, people bringing it to the house all the time. My child’s father got murdered in 2008. I tried not to grieve in front of my daughter. I turned to alcohol but I stopped the drinking and now I’m starting to eat healthy and I’m still stressed out about everything.

The participants in the child focus group noted the causes for being overweight were linked to eating junk food, having unhealthy food habits, not getting enough physical exercise, and eating too much. One of the children stated, “Eating too much junk, not eating fruit, just sitting around eating when you’re not hungry ….” They thought it was healthy to sleep and drink water.

#### Desperate for help

The parents took their children’s weight issues very seriously and have explored other avenues for assistance with their child. A few of the parents became quite emotional talking about the struggles their child faces with being overweight. One frustrated mother recalled a particularly painful event and began to cry while recounting her story. She said:

Someone sent Child Protective Services (CPS) to my house because my daughter is obese. They asked my daughter if I let her get into the refrigerator anytime. They eventually closed my case. When I take her to the doctor, I have to constantly hear about her weight. I am frustrated; I’ve tried everything and I think she is meant to be big; I just want her to be healthy. I go to so many doctors.

Many of the parents thought their doctor’s advice to them about their child’s weight was not helpful and they would like to see the physician dispense less advice and be more proactive perhaps even by giving their child appetite suppressants. For instance, one parent suggested sending her child away to a program like *The Biggest Loser* out of desperation and frustration. Another wished that the doctor would give her some medicine to make her daughter stop craving food and curb her appetite, but she felt like nothing was being done except discussing the weight.

#### Bullying

The children seemed open and willing to share their opinions on a variety of subjects, but many seemed to experience some level of distress in response to being teased or bullied, as evidenced by them speaking in lower tones, looking down, and showing less positive affect (not smiling, not as energetic) when discussing the subject. Being bullied was an issue with almost all of the children (seven of eight) involved in the study, and some of the parents shared that they were disturbed to learn that their children had been bullied because of their weight. Parents took a variety of approaches to handling their child’s bullying problems, including speaking with school officials and confronting the bullying children and their parents. One child had changed schools due to the teasing only to have their child report, “They still call me names at my new school.” However, the bullying and teasing of the children came from a variety of sources, not just their classmates, including older family, friends, and even total strangers.

The children in the study contended that teasing was the single most important motivation for them to lose weight. We asked them if they would like to be smaller, larger, or the same size. We learned that seven of the eight children wished they were smaller in size due to the bullying and did not associate being smaller with being healthier.

#### Parents’ intervention ideas

When the parents and children were asked about their ideas for creating an intervention program, what things they would most want to have in a program, and what would make them continue with a program, all of the parents stated that any type of intervention program would have to involve the whole family, so that family members can assist and support the obese child in becoming healthy. Most of the parents indicated that local churches could serve as perfect sites for the intervention, noting that many churches can provide transportation and many churches also have gyms. Transportation was mentioned as an issue, since some of the focus group participants took the city bus or were dropped off by friends in order to attend the session. A few parents mentioned involving the schools in an intervention program. Commonly, parents were not interested in having an intervention program at a clinic; however, in general, the children preferred a clinic-based intervention program. We also asked the parents about their preferred days of the week to participate in an intervention. Saturday was found to be the optimal meeting day. Ideally, the parents thought that two Saturdays per month was the appropriate time interval. One parent suggested a summer residential-type camp for the children. She stated, “Take the child out of the home for [a] special program like something in the summer, to see if there’s something I’m not doing right.”

The parents wanted an intervention program that was holistic in its approach and not one that only addressed healthy foods and weight issues. They wanted an intervention that included problematic areas such as stress management, grief counseling, physical activities, self-esteem promotion, and anger management. Some of the children in the study received counseling by different agencies and the parents thought this was beneficial to their child. The parents thought the program should be targeted to the whole family and stated that on-site childcare services should be provided for their other children who are too young to participate. One parent thought the intervention program should target the entire community.

The children’s focus groups seemed energized by the idea of exercising with their families and other children. They enjoyed the brief exercise component embedded in the focus group session using a pedometer to determine who could perform the most steps in 5 min. This was evident by their smiles and laughter during the game. The majority were also interested in learning healthy cooking strategies.

We discussed what attributes of an intervention program should not be included. One parent commented on her previous experience with another health intervention program.

I didn’t like the way they made you feel. Soon as you walked in the door, you had to weigh in. I didn’t like the condescending tone: “You’re doing it all wrong and that’s why she’s here.” It’s the way you talk to me about it. It’s somebody talking to you that weigh 80 pounds and they’ve never had a weight problem. I didn’t need more negative comments …

#### Parents distrust of technology for outreach and recruitment

The parents indicated that they would prefer to receive recruitment and advertisement materials for an intervention program through personal contact by telephone or via regular mail. While many parents had cell phones present at the focus group meeting and would check them often during the focus group, they stated they did not check texts regularly and did not use e-mail often to obtain information about attending events. The parents indicated that they receive too many text messages and would be concerned they would miss a message about an intervention program. Some parents also did not have regular Internet service, while others were mistrustful of conducting any personal type of business over the Internet.

Parents indicated that other recruitment strategies should include colorful brochures with lots of pictures mailed to their home. They thought this would help the children get excited about a program and, therefore, entice the whole family to attend the program.

Finally, excited by the idea of what she would like to participate in getting healthy, one of the children in the study asked, “When we lose weight could we get in another panel and show you?”

### Expert panel results

The qualitative analysis revealed key three themes that emerged consistently across the EP’s data: (1) health and nutritional messages, (2) physical activity, and (3) intervention strategies for target population. Verbatim comments of the EP are included to better illustrate the themes.

#### Key healthy messages

The expert panelists concurred with the focus group participants that often the messages around healthy eating; nutrition, nutritional food labeling information, and marketing information, can be confusing and contradictory to families. The EP indicated that healthy eating information is confusing for many families, not just for low-income families.

If you’re telling them eliminate sugars, sweets, and beverages and so they switch from Kool Aid to Crystal Light, and someone else is telling them artificial flavors are bad or artificial things are bad because it says artificial ….” “The same can be said for processed foods such as turkey bacon, people think that because it is turkey it is better for you, no one ever tells them it’s processed ….

The EP agreed that there is a lack of education around nutrition and healthy eating for many families. Some EP members suggested a strong promotion of the United States Department of Agriculture’s “My Plate” as the food standard. Other members of the panel suggested that when families are cooking traditional African American foods, they should be encouraged to use other spices and substitutions to reduce high-caloric intake.

The EP suggested that messages about food should be simple. Messaging should be directed to help families understand what really is “healthy.” Education about healthy eating, meal planning, shopping for groceries, and cooking was also needed to help families understand how to make the most of their limited resources to provide more healthy food options.

The EP noted that often the messages about healthy eating and lifestyle are about losing weight and focusing on the outward appearance, yet people are not as concerned with “being healthy” and taking care of their bodies and consuming foods that are good for them. The panel also suggested that health messages particularly for children should focus on health and not weight loss. For example, one member of the panel stated,

Healthy eating is not about weight reduction for a child. This is about developing appropriate behaviors that last throughout life. If healthy eating is presented to a child as weight reduction in that child’s mind, as soon as they hit whatever weight, they can go back to eating whatever it is they want to because they are not at their developmental level, able to recognize that going back to doing what you were doing in the first place is just going to lead back to weight gain.

The EP believed schools should take a more proactive role in reinforcing healthy diet, physical activity, and healthy-lifestyle information.

I think [school] is a critical place. I wouldn’t say it’s the best place. I think they need to support what the parents are trying to do at home, and so it needs to be a dual responsibility … It should be part of the educational process and it should be part of a healthy eating and healthy lifestyle throughout [life].

One of the key concerns of the expert panelists and families is that given their limited resources, choosing healthy food options can be very challenging. One of the challenges is learning to plan so that fruits and vegetables are available, but not letting them go to waste because they did not know what to do with them.

### Physical activity

The EP suggested looking at “joint-use agreements” with local school facilities or churches to provide access for physical activities that parents and children can participate in and not just team sports, where obese children are often not included. The EP also suggested that there needs to be stronger promotion of easy physical activities that can be done in the family’s homes rather than going to the YMCA or another gym. Examples of some of these activities could be dancing while you are cooking, doing leg presses with the kids or running up and down stairs in the home.

Similar to their comments about nutrition, the EP indicated that messages about physical activities should be kept simple and should promote unstructured and active play. The focus should be on activities that families and children can participate in and be successful. One suggestion was to provide families with “exercise prescriptions.” For example, the prescription could focus on one to three physical activities for the first month. On the follow up visit, the goal could be to add another physical activity or increase the time on the previous one. The goal is to make these lifelong and sustainable changes.

While the EP made recommendations for nutrition and physical activities, there was very little discussion about keeping these families motivated to create sustainable and lasting change. Many of the participants simply cautioned us about high attrition rates among this population.

#### Intervention suggestions

The EP’s opinions regarding intervention strategies were similar to the parent and child focus groups’ ideas. There was no one single strategy that emerged; however, the key elements the EP suggested included: nutrition information and cooking classes, physical activities opportunities, field trips to grocery stores to help with meal planning, psychological counseling, and periodic health assessments.

All on the panel agreed one clear goal of any intervention for these families is the inclusion of psychological counseling for the children and parents. They recognized many of the focus group participants were dealing with very difficult issues and psychological stress that was unrelated to obesity. Additionally, the panel acknowledged the parents and children were dealing with stigma associated with obesity, shame, and bullying.

Some of the EP members agreed with the parent and child focus group participants that an intervention should focus on the whole family or whole community and not just the obese child, whereas others argued for those who were obese to be involved in programs for change. Additionally, it was suggested that the intervention include parenting classes to help parents find constructive strategies to build strong and healthy families. The classes would allow parents to develop a structure for eating and meal planning along with other issues. For example, the EP indicated that parents need to assert more authority over food choices for children and be the role models for healthy behaviors and healthy lifestyles, including regular bedtimes to establish healthy sleep patterns, which impact eating routines and activity patterns. Much like the conflict between the parent and child focus groups, the EP could not agree on the best venue for the intervention but felt that the intervention should be in the community where the families are and easily accessible as transportation problems may create a barrier for many people.

Several members of the EP spoke of society not being as ready to deal with childhood obesity as it is with other conditions such as asthma or malnutrition. One panel member suggested that we are not as compelled to take action for prevention and early treatment of obesity in the same way we are for malnutrition.

## Discussion

This study examined the barriers and obstacles to healthy eating confronting low-income African American families with an obese child. Our findings are consistent with previous research on childhood obesity (10, 11). All caregivers were concerned about their child’s weight but felt hopeless in their efforts to exert any changes. Each acknowledged their child’s pediatrician had discussed the concept of healthy foods; however, they felt the messages were conflicting, which undermined their efforts. They wanted more consistent nutritional messages to help guide their choices. In contrast, the children were able to easily identify healthy foods but felt their choices were limited at home. Unfortunately, many of the families eat fast food several times per week, typically choosing foods high in fat and calories partly because it is easily accessible in low-income neighborhoods and partly because of busy work and family schedules.

Families were aware of the importance of being physically active. Most caregivers wanted their children to be physically active but were unsure of how much activity was actually needed. They identified several barriers that hindered their children’s physical exercise. The most prevalent reasons given were the caregivers’ fear of their immediate environment including dangerous neighborhoods and unkempt parks and their children’s lack of desire to be active. Many stated that their children spend most of their free time playing video games. Although all participants had a Wii™ in the home, many of the children do not take advantage of the “physical active” gaming benefits that it offers. They also commented on the lack of facilities in their neighborhood that offers physical activities in a safe environment. Typically, they had to travel to get to a park or the YMCA. Most felt their lives were too busy to take advantage of these opportunities or memberships were too costly. This perception differed from the children who stated that they were active. The children were aware of their parent’s anxiety about the dangers of the neighborhood, however, they had the desire to play outside and go to the park or YMCA. Many also expressed their aspiration to play sports, be a part of a team, and spend more time with their guardian doing physical activity. As the EP suggested, promoting unstructured play and being active while in the home might be a good alternative for families lacking resources for gym memberships or access to parks.

The theme of desperation was evident in many of the comments. Parents felt stressed by the circumstances of their daily lives. In addition, there was a lot of guilt, blame and at times shame about their children’s weight status. Some caregivers were more stressed because of the threat of Child Protective Services intervening when the child is morbidly obese as was the case with one caregiver. The majority of the children revealed that they were often bullied, which had not always been revealed to the caregiver. Many of the families lacked structure in their approach to childcare, which was evidenced by daily routines such as, nutrition, physical activity, and bedtimes.

One of the goals of this study was to determine how to design a healthy-lifestyles intervention program that will be accessible and sustainable for inner-city African American families. The researchers’ selection of only African American families from a specific geographic area and socioeconomic status was purposeful to provide an understanding of influences that shape disease perceptions. For example, spirituality is deeply tied to the African American culture. Disease initiation or recovery may be an “act of God.” The use of churches may be an option for an intervention. The use of one’s own story about a disease’s effect to provide a reference for others in the community can be a powerful tool. Many of the guardians, as well as the children, provided stories of their own or a family member with health issues related to obesity. Using this aspect of cultural sensitivity may help determine the intervention’s impact. As stated earlier, the adults as well as the children were either overweight or classified in the obesity range. An intervention that focuses on the entire family is essential for success. Understanding factors that influence a successful intervention and incorporating the two components of cultural sensitivity (surface and deep structure) will facilitate a more tailored approach to an obesity prevention and management program (14). The collaborative efforts of the families, community leaders, and researchers have potential for developing successful interventions. Ongoing and dynamic interactions and evaluations are needed to ensure that the target population’s voices are heard.

Our data suggest there are numerous options and venues to consider when designing an intervention program. Many churches often have large spaces that would serve as a safe environment to residents in the neighborhood for physical activities. This option was suggested by our families as a viable option. A key element of any intervention program should include parenting classes conducted in the neighborhood that provide more concise and consistent messaging about nutrition, meal planning, and active play. Interventions incorporating low cost options for food choices and desired flavors should be incorporated. Additionally, a counseling program is needed for both child and caregivers because many of the families were dealing with major psychological stresses that made healthy eating and lifestyles receive low priority and food was often used as a comfort. Since many of the families seemed to lack a structured home environment with children often guiding food selections and bedtimes, counseling focused on effective parenting skills and family structure should be incorporated in an intervention. The researchers found the barriers and interventions to a healthy lifestyle were not unique to low-income African American families but may be consistent with low-income families in general.

There were a number of limitations to this study. One limitation was the small sample size despite strong recruitment efforts. Although many people agreed initially to participate; however, they did not attend the session or return messages. The authors were unable to determine what barriers kept these parents from participating in the focus groups.

Recruitment and retention issues are common among this population due to transportation, child care, and scheduling conflicts. To offset the transportation issues, the focus groups were conducted in a neighborhood with ready access to bus services but this did not seem to make a difference. Recruitment may also have been problematic because some families are reluctant to discuss issues related to being overweight with people they do not know. The EP also expressed concerns about recruitment and retention strategies with regards to a successful obesity intervention. The results of this study may not be generalizable to a larger group of low-income African Americans with obese children but could provide additional insights into barriers to healthy lifestyles for low-income families with overweight and obese children.

## Conflict of Interest Statement

The authors declare that the research was conducted in the absence of any commercial or financial relationships that could be construed as a potential conflict of interest.
